# Regulating PPARG Reduces Lipid Accumulation in Microglia and Promotes Functional Recovery After Spinal Cord Injury

**DOI:** 10.1002/advs.202506313

**Published:** 2025-09-12

**Authors:** Mingran Luo, Jiayun Liu, Yunxin Su, Tao Jiang, Xincan Wu, Peng Gao, Tao Qin, Mengyuan Wu, Qingqing Li, Shujun Zhang, Baorong He, Yongxiang Wang, Xiaodong Guo, Wei Zhou, Shujie Zhao, Jin Fan, Jian Chen, Guoyong Yin

**Affiliations:** ^1^ Department of Orthopedics the First Affiliated Hospital of Nanjing Medical University Nanjing 210029 China; ^2^ Jiangsu Institute of Functional Reconstruction and Rehabilitation Nanjing Jiangsu 210029 China; ^3^ Department of Spine Surgery Wuxi Ninth People's Hospital Affiliated to Soochow University Wuxi 214061 China; ^4^ Department of Spine Surgery Honghui Hospital Affiliated to Xi'an Jiaotong University Xi'an 710054 China; ^5^ Department of Orthopedics Northern Jiangsu People's Hospital Affiliated to Yangzhou University Yangzhou 225003 China; ^6^ Department of Orthopedics Union Hospital Tongji Medical College Huazhong University of Science and Technology Wuhan Hebei 430022 China

**Keywords:** atorvastatin, lipid droplet, microglia, PPARG, single‐cell sequencing, spinal cord injury

## Abstract

Spinal cord injury (SCI) substantially affects functional capacity and the immune system plays a crucial role in recovery. Examining alterations in microglia metabolism can lead to improved repair mechanisms; however, the molecular subtyping of microglia lacks consensus. In this study, the effects of SCI on macrophages and microglia in mice are investigated to identify tailored therapeutic targets and interventions for patients with SCI. Macrophages infiltrate the spinal cord shortly after injury; however, infiltration decreases over time. Microglial phagocytosis of myelin debris is associated with increased lipid accumulation. Macrophage deletion improves outcomes, whereas microglial deletion worsens them. The PLIN2+ microglia subtype in lipid droplet formation shows abnormal activation of the Pparg signaling pathway compared with that with other subtypes. PPARG promotes lipid metabolism and recovery, and atorvastatin (a PPARG agonist) reverses altered metabolic processes. Macrophages and microglia play complex roles in SCI. Targeting PPARG and its agonists is a promising therapeutic approach for SCI.

## Graphical abstract

Macrophages infiltrate the spinal cord post‐injury, decreasing over time. Microglia phagocytose myelin debris, increasing lipid accumulation. Macrophage deletion improves outcomes, while microglial deletion worsens them. The LD+ microglia subtype shows abnormal Pparg signaling. PPARG promotes lipid metabolism and recovery, and atorvastatin reverses altered metabolic processes. Targeting PPARG and its agonists offers a promising therapeutic approach for spinal cord injury.

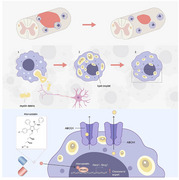

## 1 Introduction

Spinal cord injury (SCI) encompasses structural or functional impairments of the spinal cord that stem from various causes. This damage causes considerable physical, emotional, and financial burden, substantially diminishing the quality of life of patients. Approximately 17 000 new cases are reported annually, with a prevalence of 54 new SCI cases per million individuals in the United States and ≈6.4 cases per million in China.^[^ ^1^ ^]^ Patients with SCI often have a poor prognosis and endure lifelong disability due to the limited regenerative capabilities of the central nervous system (CNS). Furthermore, the immune response may hinder recovery post‐SCI, contributing to secondary injury, persistent inflammation, and glial scar formation.^[^ ^2^ ^]^ The aftermath of SCI, marked by neuronal loss, myelin destruction, and vascular damage, triggers inflammation and initiates secondary neuropathological events, such as scar formation. These events hinder axonal regeneration and overall functional recovery.^[^ ^3^ ^]^ Microglia are universally found in many CNS diseases. However, the molecular subtyping of microglia lacks consensus.

The accumulation of lipid substances after SCI leads to the appearance of lipid droplets (LDs). LDs are organelles involved in the storage and utilization of lipids. They are increasingly recognized as dynamic, versatile organelles, playing active roles in various physiological and pathological processes, such as neurodegeneration, tumorigenesis, and embryonic development. LDs feature a core of neutral lipids encompassed by a phospholipid monolayer and various proteins. The molecular mechanism of LD deposition is still unknown. Peroxisome proliferator‐activated receptor gamma (PPARG) belongs to a group of nuclear receptors that regulate reproduction, metabolism, development, and immune responses. Upon activation by a specific agonist, these receptors dimerize and are translocated to the nucleus. In the nucleus, they function as agonist‐dependent transcription factors, controlling gene expression by binding to specific promoter sequences of target genes. PPARG exhibits diverse biological functions and is crucial in metabolism regulation, inflammation control, and immune process modulation.^[^ ^4^ ^]^ After injury, the spinal cord tissue contains abundant lipid components, which are vital to the myelin sheath, neurotransmission, and axon growth. SCI is accompanied by disintegration of the myelin sheath, resulting in the accumulation of lipid components that may affect neuronal survival. Moreover, during oxidative stress, intracellular lipids are readily peroxidized into toxic substances, which, if not eliminated quickly, can promote cell apoptosis or ferroptosis. The aim of this study was to investigate genetic alterations and potential metabolic disparities, and explore which cells play a key role in accumulating lipids and the impact of their intervention on recovery from SCI.

## 2 Results

### 2.1 Abnormal Lipid Accumulation with SCI

**Figure 1:**
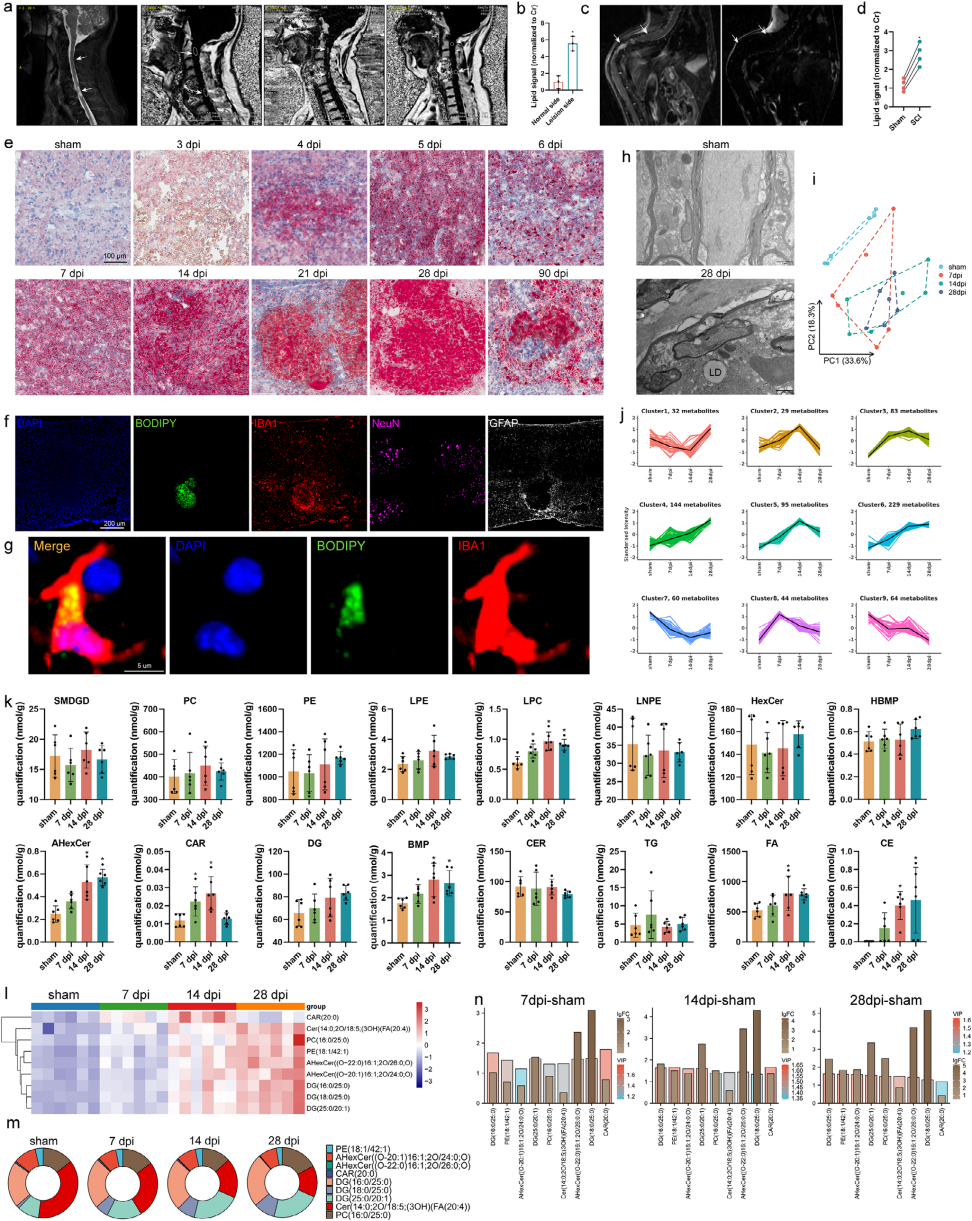
Microglia in SCI accumulate LDs. a,b) Spinal cord DTI of participants with SCI showing lipid peaks on the lesion and contralateral normal sides. Representative T2 of one participant and DTI images and statistics for all participants (n = 3). c,d) Spinal cord DTI of SCI mice showing lipid peaks on the lesion and contralateral normal sides. Representative T2 and DTI images of one participant and statistics for all participants (n = 4). e) Oil Red O staining results and analysis (scale bar: 100 µm). f) Spinal cords from SCI mice stained with BODIPY+ (LDs), IBA1+ (microglia), NeuN (neurons), and GFAP (astrocytes). g) Representative confocal images of BODIPY+ (LDs) and IBA1+ microglia in mice with SCI. h) Electron microscopy images of sham and SCI mice. i) Principal component analysis showing significant differences in lipid composition before and after spinal cord injury. j) Changing trend of different lipid components with injury time, among which 373 lipid components gradually increased. k, Total cholesteryl esters (CE), ceramides (Cer), diacylglycerols (DAG), free fatty acids (FFAs), hexosyl ceramides (HCer), lysophosphatidylcholine (LPCs), lysophosphatidylethanolamine (LPE), phosphatidylcholine (PC), phosphatidylethanolamine (PE), and triglycerides (TAGs) measured by direct infusion of MS from sham mice at 7, 14, and 28 dpi. l) Heat map of significant increase in lipid composition, except for Arachidyl carnitine (CAR) (20:0). m) Pie chart of the proportion and change in each component. n) Bar chart of the differences in changes in the nine substances between the two groups. Data are shown as mean ± SD and p‐values were determined using a two‐tailed unpaired *t*‐test. ^*^ *p *<0.05 compared with the SCI or sham groups. DTI, diffusion tensor imaging; SCI, spinal cord injury; MS, mass spectrometry; LD, lipid droplet.

Abnormal lipid accumulation in the spinal cord of individuals with SCI was investigated using diffusion tensor imaging (DTI). An apparent increase in the lipid signal was detected in the foci of participants with SCI (n = 3) compared with that in normal spinal cords, indicating an abnormal accumulation of lipids in the injured spinal cord (**Figure** **1**a,b). These clinical observations were confirmed in a classical SCI model. To detect lipids in vivo, lipid levels were quantified in the mouse spine using animal DTI. A significant increase in lipid signals was detected in the spinal cords of SCI mice 4 weeks after SCI (Figure 1c,d). When sections from each group were stained with Oil Red O, lipids gradually accumulated over the injury and reached a peak value at 28 dpi (Figure 1e; Figure S1b, Supporting Information).

Histological staining of IBA1+ microglia with BODIPY, a dye that specifically labels neutral lipids and is commonly used to detect LDs, showed that LDs were abundant in the spinal cord of SCI mice (Figure 1).^[^ ^5^ ^]^ Therefore, subsequent analyses were focused on this region. LDs were primarily found in microglia; however, they were absent in other cell types (Figure 1f). The percentage of BODIPY + IBA 1+ microglias in the SCI group was over fourfold higher in normal microglia and LDs were significantly larger in SCI microglias (Figure S1a,c, Supporting Information). Microglial LDs were also immunoreactive for the LD surface protein, perilipin 2 (PLIN2; Figure S7a,b, Supporting Information). We analyzed the structural differences between normal and SCI microglias using transmission electron microscopy to determine their cytoplasmic content. Characteristic LDs were observed in SCI microglias, but rarely in normal microglias (Figure 1h).

Lipidomic profiling was performed to analyze the lipid components of the spinal cord. SCI increased downstream cholesterol esters (CEs), ceramide (Cer), and diacylglycerol (DAG) metabolites without significantly altering other lipid classes.^[^ ^6^ ^]^ The CE, and fatty acid (FA) lipid levels were significantly higher in SCI than those in normal controls (Figure 1k). Principal component analysis (PCA) showed significant differences in the lipid composition before and after SCI (Figure 1i). Moreover, the heat map revealed the changes in lipid composition between the groups after SCI (Figure S1f–i, Supporting Information). The line chart shows the changing trend of different lipid components with injury time, among which 373 lipid components gradually increased, consistent with the staining results (Figure 1j). The differences were analyzed for each group. The uptake map showed nine lipid components with significant differences between groups (Figure S1e, Supporting Information). Furthermore, the heat map shows a substantial increase in lipid composition, excluding arachidyl carnitine (20:0) (Figure 1l). The pie chart shows the proportion and change in each component (Figure 1m). The bar chart shows the differences in changes in the nine substances between the two groups (Figure 1n). These data demonstrate that abnormal lipid accumulation in the spinal cord is a significant pathological feature of SCI.

### 2.2 Myelin Phagocytosis Induces LD Formation in Microglia

**Figure 2:**
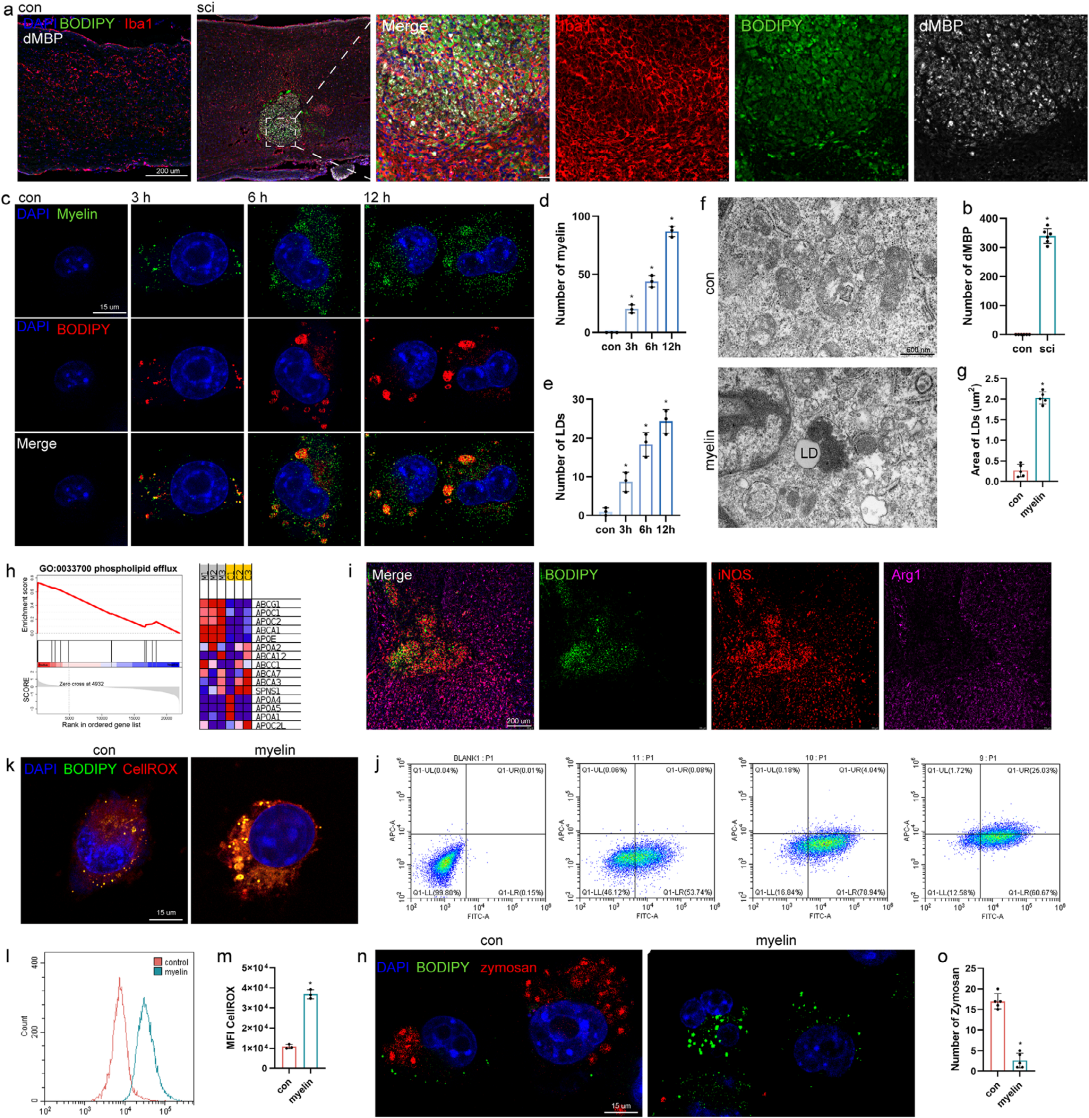
Myelin phagocytosis induces lipid droplet formation in microglia. a) IF staining results for IBA1 (red), BODIPY (green), dMBP (white), and DAPI (blue). b) Quantification of dMBP+. c–e) Confocal images (c), and quantification of myelin+(d) and BODIPY+(e) in cells treated with myelin (20 µg mL^−1^) for 3, 6, and 12 h. f) Electron microscopy image showing lipid accumulation in myelin‐treated cells. g) Quantification of average BODIPY+ lipid droplet size. h) Transcriptome detection of differential genes mainly enriched in the phospholipid efflux process and heat map of gene expression involved in the process i on the right. i) IF staining for iNOS (red), BODIPY (green), and ARG1 (purple) in the spinal cord. j) Flow cytometry histogram of myelin+ and iNOS+ fluorescence in cells. k) CellROX fluorescence in cells treated with PBS or myelin (5 µg mL^−1^) for 24 h. Representative confocal images of CellROX+ signal in cells. l,m) Flow cytometry histogram (k) and quantification (l) of CellROX fluorescence in cells. n,o) Confocal images (n) and quantification (o) of BODIPY+ and zymosan+ in cells treated with myelin (20 µg mL^−1^) for 24 h. Data are shown as mean ± SD and p‐values were determined using a two‐tailed unpaired *t*‐test. ^*^ *p* <0.05 compared with the myelin or con groups. dMBP, degraded myelin basic protein.

In addition to numerous LDs, immunofluorescence staining showed that an accumulation of degraded myelin basic protein (dMBP) exists in the SCI area. Phagocytosis and clearance of dMBP by microglias was suggested to lead to lipid accumulation (**Figure** **2**a,b). Myelin was extracted and this finding was verified in vitro. Myelin was added to the cell culture, and phagocytosis and lipid accumulation were observed. Over time, myelin phagocytosis by microglias and lipid accumulation increased (Figure 2c–e). Electron microscopy revealed lipid accumulation following myelin phagocytosis (Figure 2f,g). This confirmed that myelin phagocytosis leads to lipid accumulation. Phospholipid efflux pathways have also been observed in microglia after myelin co‐culture. The ABCG1, ABCA1, and APOC1 levels in this pathway did not increase after myelin stimulation (Figure 2h).

Immunofluorescence staining revealed numerous anti‐inflammatory and pro‐inflammatory microglia surrounding the injured area (Figure 2i). Moreover, primary microglia with myelin sheaths were stimulated in vitro and flow cytometry was used to demonstrate that microglia gradually transformed into the pro‐inflammatory type with phagocytosis (Figure 2J). After myelin co‐culture, the cells accumulated more LDs, and the level of reactive oxygen species (ROS) significantly increased, which may be detrimental to further recovery from SCI (Figure 2l,m). LDs and myelin‐containing microglia were introduced to assess whether their phagocytic activity is altered in cells with myelin. The cells were exposed to zymosan particles derivatized with pHrodo, a fluorescent indicator of cellular uptake in acidic compartments and lysosomes. Myelin decreased phagocytosis. However, zymosan particles were mainly found in the BODIPY cell population and, to a significantly lesser extent, in LD‐rich BODIPY+ cells (Figure 2n,o).

### 2.3 Single‐Cell RNA Sequencing (scRNA‐Seq) of CD45+ and BODIPY+/‐ SCI Mice

**Figure 3:**
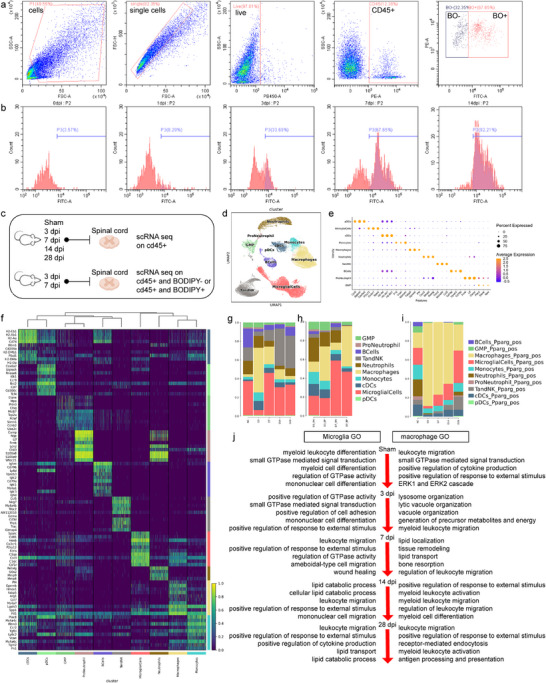
Single‐cell RNA sequencing revealed abnormalities in lipid metabolism and upregulation of Pparg+ microglia. a,b) Flow sorting scheme for the isolation of BODIPYlo (LD‐low) and BODIPYhi (LD‐high) CD45lo cells from the spinal cords of SCI mice. c) Schematic of scRNA‐seq using flow‐sorted samples from SCI or control mice. d) Unsupervised clustering of cell populations. e) Dot plot depicting selected differentially expressed genes for each cluster. Dot size corresponds to the percentage of gene expression in each cluster; color represents the average gene expression level. f) Heat map representing gene expression of the top 10 DEGs in each cluster. Gene expression levels were color‐coded, ranging from dark blue (low expression levels) to yellow (high expression levels). g,h) Cell percentage dynamics at different times in the injured mouse spinal cord. i) Pparg+ cell percentage dynamics at different time points in the injured mouse spinal cord. j) GO biological process terms associated with the top DEGs between the time points for microglia (left) and macrophages (right). GO, Gene Ontology; SCI, spinal cord injury.

Flow cytometry was performed at different time points after injury to determine the content and proportion of lipid‐positive cells after SCI. The number of lipid‐positive cells gradually increased, reaching 93% at 14 days (**Figure** **3**a,b). ScRNA‐seq was performed in SCI mouse models to detect differences in lipid accumulation at different time points and in various cell types after SCI. To further analyze the cell components and specific subpopulations, cells were isolated from the spinal cord of SCI mice based on CD45+ and BODIPY+. The differences in cell transcription between low and rich LDs were examined (Figure 3c). In total, 66526 single‐cell transcriptomic profiles were obtained after filtering low‐quality nuclei (Figure 2a). Cluster analysis identified 10 major cell types hallmarked by key representative marker genes (Figure 3d). Microglia were defined by the expression of the canonical markers: P2ry12, Cx3cr1, and Sparc (Figure 3e,f).

Furthermore, cell population analysis revealed considerable differences between SCI and non‐SCI controls, including macrophage infiltration and microglial activation (Figure 3g,h).

Although lipid accumulation was observed in both microglia in the above experiments, Gene Ontology (GO) enrichment analysis of the scRNA‐seq data showed that lipid transport‐ and metabolism‐related pathways were markedly activated in microglia at 28 dpi, yet only moderately activated in macrophages (Figure 3j).

PLIN is the most prevalent LD protein, playing a pivotal role in regulating lipid storage, especially by shielding LDs from lipase activity. Five distinct isoforms of PLIN (PLIN1 to PLIN5) exist, each displaying unique patterns of tissue distribution and functional characteristics. Notably, PLIN1 and PLIN4 are predominantly expressed in adipose tissue, whereas PLIN2 and PLIN3 are widely expressed throughout the body. Elevated PLIN2 levels in the heart result in substantial lipid accumulation in the myocardium, suggesting its importance in stabilizing LDs. Research in hepatocytes has further indicated that increased PLIN2 expression protects against autophagy, while its reduction stimulates triglyceride catabolism through autophagy. Additionally, in vitro studies have demonstrated that the absence of PLIN2 reduces lipid accumulation by disrupting the lipase barrier associated with LDs, leading to enhanced lipolysis. Moreover, recent research has emphasized PLIN2 involvement in targeted lipophagy of LDs.^[^ ^7^ ^]^

Targeting foam cell formation processes is the best approach by stimulating the lipid efflux transporters ABCA1 and ABCG1 directly or indirectly via PPARG. Especially, as stimulating the reversed cholesterol transport is efficient in restoring the capacity to remyelinate lesioned tissues.^[^ ^8^ ^]^

The expression rates of Pparg and Plin2 gradually decreased in macrophages and progressively increased in microglia, suggesting that lipid accumulation is more closely related to microglia (Figure 3i). Differential expression between mice with SCI and controls was analyzed using scRNA. A panel of upregulated lipid‐sensing, transport, storage, and hydrolysis genes, including APOE and ABCA1, were identified. To quantitatively infer and analyze the cell communication networks that emerge post‐SCI, microglia and macrophage were computationally isolated from the scRNA‐seq dataset clusters. CellChat was used to decode complex signaling patterns involving soluble and membrane‐bound ligand‐receptor interactions. The chord plot in Figure S5 (Supporting Information) illustrates the autocrine and paracrine signaling interactions with color‐coded ligand‐receptor interaction scores. Macrophages and microglia highly interacted at 3 and 14 dpi, respectively, indicating the timing difference when cells do not play a primary role. This observation suggests that immune cell infiltration during the SCI phase significantly affects the spinal cord microenvironment. In addition, differences were observed in transcription factors that played key roles in the two cell types at different time points (Figure S6, Supporting Information).

Furthermore, microglial LDs were immunoreactive for the LD surface protein, PLIN2 (Figure S7a, Supporting Information). PLIN2 was detected in microglia in spinal cord sections and PLIN2+ IBA1+ microglia were mainly confined to the damaged areas (Figure S7b, Supporting Information). In myelin‐co‐cultured cells, the PLIN2 protein was mostly concentrated around lipids. Moreover, the PLIN2 content was low in cells without lipids, suggesting a significant correlation between PLIN2 and lipid content. The number of PLIN2+ microglia gradually increased, while the number of PLIN2+ macrophages gradually decreased, suggesting that microglia is crucial in lipid accumulation in SCI (Figure S7c, Supporting Information). In the subpopulation analysis, the PIN2+ positivity rate in the lipid‐binding microglia (LBM) subgroup was higher (Figure S7d, Supporting Information), indicating that the LBM subgroup might be the main subgroup of accumulated lipids.

### 2.4 Molecular Profiling of Microglia with SCI

**Figure 4:**
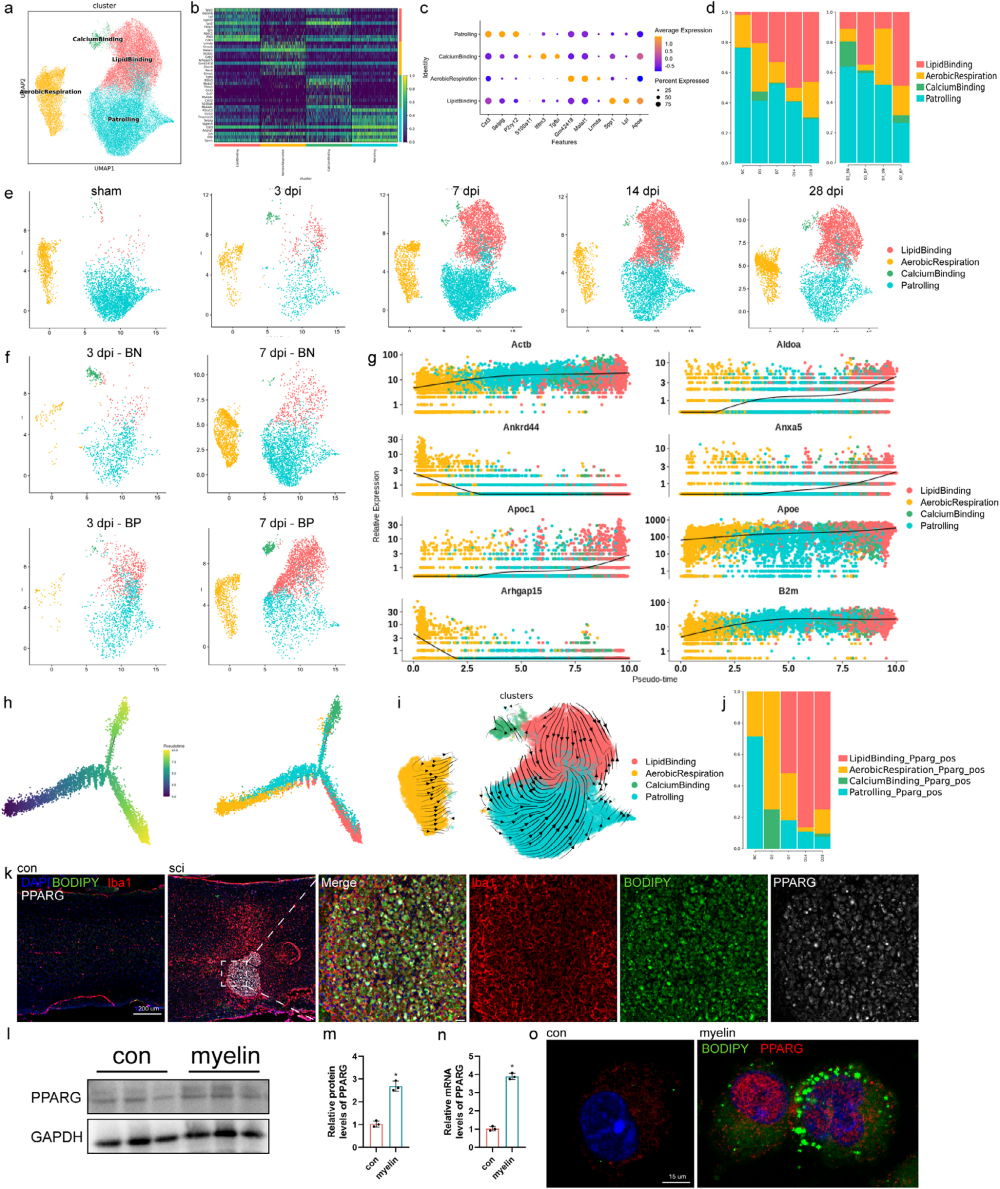
Molecular profiling of in microglia with SCI. a) Unsupervised subclustering of astrocytes using scRNA‐seq data. b) Heatmap representing gene expression of the top 10 DEGs in each cluster. Gene expression levels were color‐coded, ranging from dark blue (low expression levels) to yellow (high expression levels). c) Dot plot depicting selected differentially expressed genes for each cluster. Dot size corresponds to the percentage of gene expression in each cluster; color represents the average gene expression level. d) Number of cells in each subcluster. e,f) Uniform manifold approximation and projection (UMAP) of each sample. g–i) Transcriptome pseudotime and RNA velocity comparison of cells before and after SCI in mice. j) Pparg+ cell percentage dynamics at different time points in microglia. k) IF staining results for IBA1 (red), BODIPY (green), PPARG (white), and DAPI (blue). l) The levels of PPARG protein were detected using WB at various time points after spinal cord injury. m) The level of PPARG protein was detected by WB after myelin co‐incubation. n,o) Quantitative analysis of the WB results. p) *Pparg* mRNA levels in cells after myelin co‐incubation. q) Confocal images of PPARG in cells treated with myelin (20 µg mL^−1^) for 24 h, SCI. spinal cord injury. Data are shown as mean ± SD and p‐values were determined using a two‐tailed unpaired *t*‐test. ^*^ *p *<0.05 compared with the myelin or con groups.

Subsequently, whether abnormal lipid accumulation in microglia was related to their particular subtype was investigated. The “lipid binding” subcluster displayed high expression of markers for “lipid metabolism.” More importantly, the LB subgroup did not exist in the sham group yet gradually appeared after injury and was more abundant in the LD+ group, suggesting that the LB subgroup may be a functional subgroup after SCI, and its function is significantly related to lipid metabolism (**Figure** **4**a–f). Transcriptome pseudotime and RNA velocity also suggested that the LB subgroup was terminally differentiated (Figure 4g,i). Significantly more PPARG‐positive cells were observed in this subgroup than that in the other subgroups, indicating that PPARG plays a key role in lipid metabolism (Figure 4j). Consistent with the scRNA‐seq results, immunofluorescence staining confirmed the high expression of PPARG after spinal cord injury (Figure 4k). Elevated Pparg expression in myelin‐co‐incubated primary microglial samples was verified using immunoblotting (Figure 4l,m) and immunofluorescence (Figure 4o). RT‐qPCR analysis further confirmed the increased expression of *Pparg* in the SCI mice (Figure 4n). These data indicate disease‐associated astrocytic lipid metabolism dysfunction in the spine and identify *PPARG* as a critical gene involved in this process. Therefore, a subgroup related to lipid metabolism in the damaged region was defined as LBM.

### 2.5 Lipid Accumulation is More Closely Associated with Microglia

C57BL/6JGpt‐H11^em1Cin(CAG‐LSL‐tdTomato)^/Gpt mice were used to explore which cells colocalize more with lipid accumulation. The microglia and LDs were more colocalized at the later stage of injury when lipid accumulation was at its maximum, suggesting that the long‐term accumulation of lipids after SCI was more closely related to microglia (Figure S2a–d, Supporting Information). Using xenografted mice, the location distribution and LD colocalization of macrophages and microglia were analyzed. The number of infiltrated CD45.1+ macrophages and LD colocalization was lower than those of IBA1+ microglia (Figure S2e–h, Supporting Information).

Diphtheria toxin subunit a (DTA) mice were used to delete macrophages and microglia.^[^ ^9^, ^10^, ^11^ ^]^ The number of LDs decreased to different degrees. However, significant differences were found in the functional recovery after injury. Functional recovery worsened after microglial removal and slightly improved after macrophage removal (Figure S3a–i, Supporting Information). This suggests that numerous infiltrating macrophages may adversely affect the recovery from SCI, although they may reduce lipid accumulation. Deletion of microglia may also lead to a reduction in lipid accumulation; however, it may also be important in the recovery process of SCI. The same cell deletion was performed with phosphate liposomes and PLX5622 and results were consistent with those of the previous experiment (Figure S4a–j, Supporting Information).^[^ ^12^, ^13^ ^]^ Deleting either cell type led to reduced lipid accumulation, suggesting that both cell types play a role in lipid accumulation. However, large macrophage infiltration can lead to dysfunction in functional recovery, whereas microglia is critical in the recovery process.

### 2.6 Effects of Overexpression of Pparg on Cells

**Figure 5:**
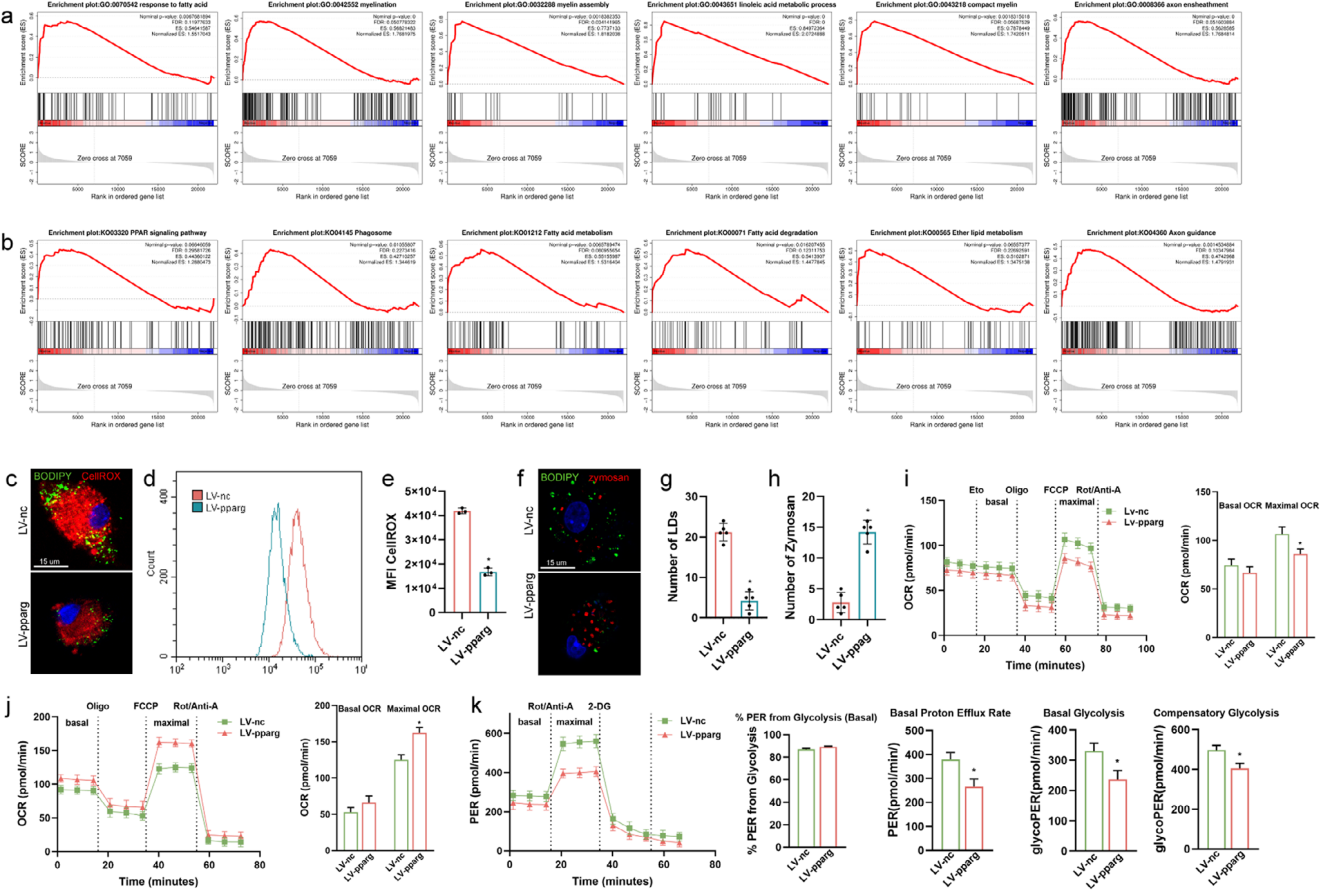
Pparg Overexpression improves SCI by reducing LDs. a,b) Representative GO (response to fatty acid, myelination, myelin assembly, linoleic acid metabolic process, compact myelin, axon ensheathment) and pathways (PPAR signaling pathway, phagosome, fatty acid metabolism, fatty acid degradation, ether lipid metabolism, axon guidance) enriched in the identified genes as determined by GSEA (normal *p* <0.05). c) Representative images of BODIPY+ (LDs) and IBA1+ microglia in SCI mice. d) Electron microscope images of the LV‐pparg and LV‐nc spinal cords. e,f) Quantification of BODIPY+ LD numbers (e) and the percentage of BODIPY+ IBA1+ cells (f) in the spinal cord. n = 6 mice per group. g) Quantification of average BODIPY+ LD size. h) CATWALK analysis on day 28 post‐injury demonstrated better functional recovery in Pparg‐overexpressing mice (n = 6). i) The footprints quantification of mice walking 28 days after SCI. j) Pole‐climbing tests on day 28 post‐injury demonstrated better functional recovery in Pparg‐overexpressing mice (n = 6). k) Rotarod tests on day 28 post‐injury demonstrated better functional recovery in Pparg‐overexpressing mice (n = 6). l) BMS scores during 28 days of recovery after SCI demonstrate better functional recovery in Pparg‐overexpressing mice (n = 6). m) CellROX fluorescence in cells treated with LV‐pparg or LV‐nc. Representative confocal images of CellROX+ signal in cells. n,o) Flow cytometry histogram (n) and quantification (o) of CellROX fluorescence in cells. p–r) Confocal images (p), and quantification of BODIPY+ (q) and zymosan+ (r) in cells treated with LV‐pparg or LV‐nc. s–u) Seahorse respirometry and measurement of oxygen consumption rate (OCR), extracellular acidification rate (ECAR), and Palmitic acid metabolism in LV‐pparg and LV‐nc cells. OCR or ECAR quantification is displayed on the right. Data are shown as mean ± SD and p‐values were determined using a two‐tailed unpaired *t*‐test. ^*^ *p* <0.05 compared with the LV‐pparg or LV‐nc groups. LD, lipid droplet; BMS, Basso Mouse Scale; LV, left ventricle; nc, negative control; OCR, oxygen consumption rate; SCI, spinal cord injury.

The left ventricle (LV) was used to explore the effects of Pparg overexpression in vivo and in vitro. Transcriptome sequencing was performed on cells overexpressing PPARG, and the genes that changed after overexpressing PPARG were mainly enriched in the “response to fatty acid,” “myelination,” “myelin assembly,” “linoleic acid metabolic process,” “compact myelin,” and “axon ensheathment” biological processes, and “PPAR signaling pathway,” “phagosome,” “fatty acid metabolism,” “fatty acid degradation,” “ether lipid metabolism,” and “axon guidance” signaling pathways (**Figure** **5**a,b)

Pparg overexpression in vivo was consistent with that in vitro, both of which reduced the formation of LDs, reduced the level of ROS, and restored the phagocytic function of microglia (Figure 5c–e). The number of LDs significantly decreased after Pparg overexpression. The phagocytic function was also recovered, and phagocytosis of yeast polysaccharides significantly increased (Figure 5f–h). To assess the metabolic alterations in microglia and their response to myelin phagocytosis, Seahorse technology was used to analyze the cellular metabolic status. Pparg overexpression led to a significant reduction in oxygen consumption rate (OCR; Figure 5i). In contrast, the proton efflux rate (PER) significantly increased (Figure 5j). Furthermore, lipid metabolism in LV‐Pparg cells was substantially reduced, potentially due to lipid depletion within these cells (Figure 5k).

### 2.7 Effects of Overexpression of Pparg and PPARG‐cKO on Functional Recovery in Mice

**Figure 6:**
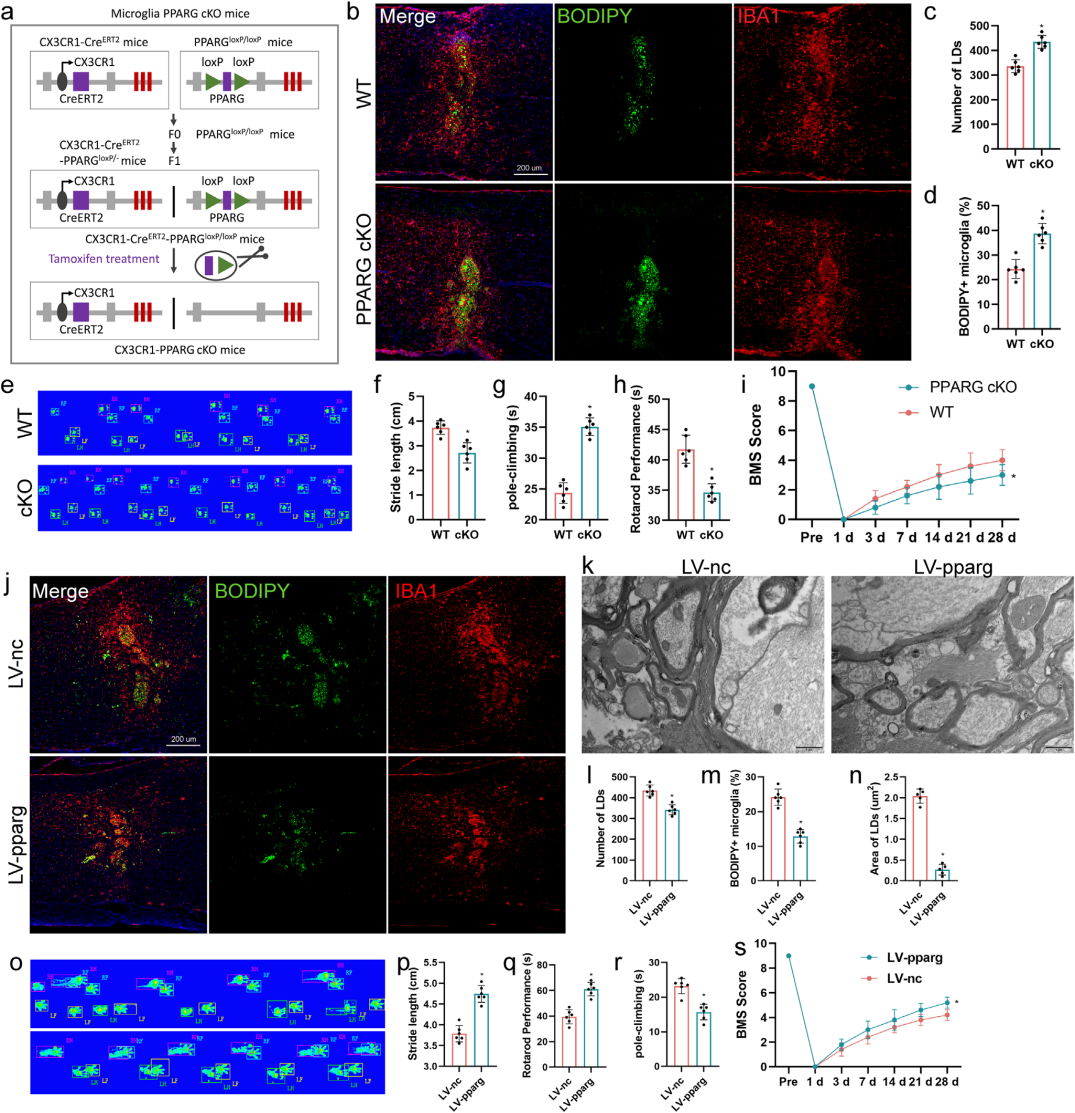
PPARG is required for the lipid metabolism. a) cKO of PPARG in microglia was achieved by crossing CX3CR1‐CreERT2 mice with PPARG‐loxP mice. b–d) Representative images (b) of BODIPY+ (LDs) and IBA1+ microglia in cKO and WT mice. Quantification of BODIPY+ LD numbers (c) and the percentage of BODIPY+ IBA1+ cells (d) in the spinal cord. n = 6 mice per group. e) CATWALK analysis on day 28 post‐injury demonstrated worse functional recovery in Pparg‐cKO mice (n = 6). f) The footprints quantification of mice walking 28 days after SCI. g) Pole‐climbing tests on day 28 post‐injury demonstrated worse functional recovery in Pparg‐cKO mice (n = 6). h) Rotarod tests on day 28 post‐injury demonstrated worse functional recovery in Pparg‐cKO mice (n = 6). i) BMS scores during the 28 days of recovery after SCI demonstrated worse functional recovery in Pparg‐cKO mice (n = 6). BMS, Basso Mouse Scale; cKO, conditional knockout; LD, lipid droplet; SCI, spinal cord injury. Data are shown as mean ± SD and p‐values were determined using a two‐tailed unpaired *t*‐test. ^*^ *p *<0.05 compared with the pparg or con groups.

An astrocyte‐specific conditional knockout (cKO) of Pparg was generated by crossing CX3CR1‐CreERT2 mice with Pparg‐floxP mice (**Figure** **6**a). Microglia‐specific PPARG cKO mice exhibited a significant increase in LD accumulation in microglia after SCI (Figure 6b–d). Hindlimb locomotor function was rigorously evaluated in an open field on days 1 and 3, and weekly for up to 4 weeks following injury. Pparg‐cKO mice showed worse gait scores after injury. Notably, the Basso mouse scale (BMS) scores of Pparg‐cKO mice were significantly lower than those of WT mice, implying a worse prognosis for motor recovery following SCI in LV‐Pparg mice (Figure 6i). Pparg‐cKO SCI mice displayed worse hind paw placement than WT mice (Figure 6e,f). Furthermore, a shorter duration of the rotarod performance test (Figure 6g) and longer time required for the pole‐climbing test (Figure 6h) indicated that motor function recovery in Pparg‐cKO mice was worse than that in WT mice. These data demonstrate that microglial PPARG mediates the active transport of lipids during SCI. Immunofluorescence and electron microscopy results showed less lipid accumulation in LV‐Pparg mice after SCI (Figure 5j–n). Hindlimb locomotor function was rigorously evaluated in an open field on days 1 and 3, and weekly for up to 4 weeks following injury. Within 24 h of SCI, all injured mice exhibited a BMS score of 0 or 1, indicating a complete loss of motor function. Pparg‐overexpressing mice showed a better gait after injury. The BMS scores of LV‐Pparg mice were significantly higher than those of LV‐nc (negative control) mice, implying a better prognosis for motor recovery following SCI in LV‐Pparg mice (Figure 5s).

The CatWalk apparatus to quantify the gait dynamics to further understand locomotor kinematics beyond the BMS scores. Four weeks after SCI, mice capable of placing the plantar surface of their hind paws on a flat surface were selected for the evaluation. The stepping pattern, a crucial indicator of overall motor coordination, monitors the sequential placement of the paws during a step cycle. Compared with that in sham mice, stride length (the total distance between consecutive placements of the same paw) was significantly shortened in all injured mice. LV‐Pparg SCI mice displayed significantly improved hind paw placement compared with that of LV‐nc mice (Figure 5o,p). Furthermore, the longer duration of the rotarod performance test (Figure 5q) and shorter time required for the pole climbing test (Figure 5r) indicated that motor function recovery in LV‐Pparg mice was better than that in LV‐nc mice.

### 2.8 Atorvastatin Activates PPARG to Promote Lipid Metabolism and Functional Recovery

**Figure 7:**
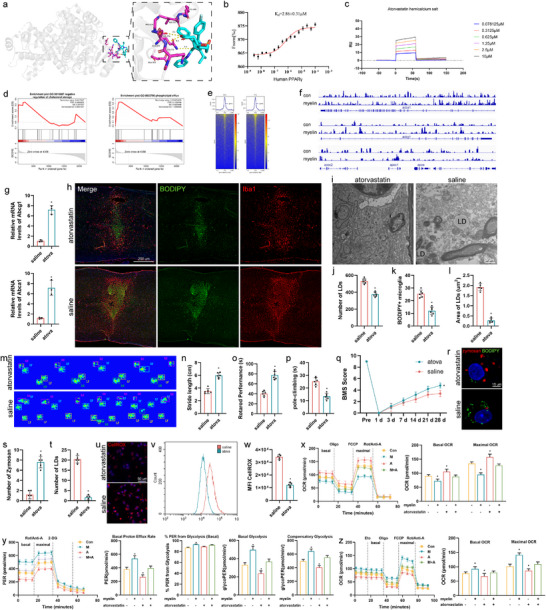
Atorvastatin activates PPARG to promote lipid metabolism and functional recovery. a) The 3D image reveals that atorvastatin binds to the binding pocket of PPARG. b) Surface plasmon resonance (SPR) analysis of the binding affinity of atorvastatin to the PPARG protein. c) Microscale thermophoresis (MST) of atorvastatin. PPARG cells were incubated with increasing concentrations of atorvastatin. d) Representative pathways enriched in the identified genes as determined by GSEA (normal *p <* 0.05). e) CUT&Tag peak heatmap showing PPARG binding loci in microglia with treatment of myelin. f) Integrative Genomics Viewer (IGV) tracks showing PPARG CUT&Tag at *Abca1, Abcg1, Apoc1, Apoc2*, and *Apoe* gene loci. g) *Abca1* and *Abcg1* mRNA levels in cells after atorvastatin treated. h) Representative images of BODIPY+ (LDs) and IBA1+ microglia in the SCI mice. i) Electron microscope images of the LV‐pparg and LV‐nc spinal cords. j,k) Quantification of BODIPY+ LD numbers (j) and percentage of BODIPY+ IBA1+ cells (k) in the spinal cord. n = 6 mice per group. l) Quantification of average BODIPY+ LD size. m) CATWALK analysis on day 28 post‐injury demonstrated better functional recovery in atorvastatin‐treated mice (n = 6). n) The footprints quantification of mice walking 28 days after SCI. o, Pole‐climbing tests on day 28 post‐injury demonstrated better functional recovery in atorvastatin‐treated mice (n = 6). p) Rotarod tests on day 28 post‐injury demonstrate better functional recovery in atorvastatin‐treated mice (n = 6). q) BMS scores during 28 days of recovery after SCI demonstrated better functional recovery in atorvastatin‐treated mice (n = 6). r–t) Confocal images (r) and quantification of zymosan+ (s) and BODIPY+ (t) in cells treated with atorvastatin or saline. u) CellROX fluorescence in cells treated with atorvastatin (5 µg mL^−1^) or saline. Representative confocal images of CellROX+ signal in cells. v,w) Flow cytometry histogram (v) and quantification (w) of CellROX fluorescence in cells. x–z) Seahorse respirometry and measurement of oxygen consumption rate (OCR), extracellular acidification rate (ECAR), and Palmitic acid metabolism in atorvastatin or myelin cells. OCR or ECAR quantification is displayed on the right. Data are shown as mean ± SD and p‐values were determined using a two‐tailed unpaired *t*‐test. ^*^ *p *<0.05 compared with the atorvastatin or saline groups. BMS, Basso Mouse Scale; LD, lipid droplet; OCR, oxygen consumption rate; SCI, spinal cord injury.

The top 200 compounds in the library of MCE‐marketed drugs targeting PPARG that could cross the blood‐brain barrier were selected. Twenty‐one compounds were obtained, and tested for CCK8 toxicity and their effects on lipid accumulation (Figure S8a,b, Supporting Information). Molecular docking revealed that atorvastatin can target PPARG to exert its effect (**Figure** **7**a). Further tests using surface plasmon resonance (SPR) and microscale electrophoresis (MST) both verified that atorvastatin could effectively bind to PPARG (Figure 7b,c). Atorvastatin effectively reduced lipid accumulation and was safe to use. Transcriptome sequencing was performed on atorvastatin‐treated cells. The genes upregulated after atorvastatin treatment were mainly enriched in the “negative regulation of cholesterol storage” and “phospholipid efflux” biological process (Figure 7d). The cut‐tag results showed that after atorvastatin treatment, the transcription level of Pparg significantly increased, and downstream Abca1 and Abcg1 were substantially regulated by atorvastatin (Figure 7e,f). Further verification showed that the *ABCA1* and *ABCG1* mRNA levels in the atorvastatin group significantly increased (Figure 7g). Immunofluorescence and electron microscopy revealed reduced lipid accumulation in atorvastatin‐treated mice after SCI (Figure 7h–l). Atorvastatin‐treated mice showed better gait after injury. Notably, the BMS scores of atorvastatin‐treated mice were significantly higher than those of control mice, implying a better prognosis for motor recovery following SCI (Figure 7q). Atorvastatin‐treated SCI mice displayed significantly improved hind paw placement compared with that of control mice (7m,n). Similarly, atorvastatin‐treated mice performed better in the rod‐climbing and spinning experiments (Figure 7o,p). These results indicate a better prognosis for motor recovery following SCI in the atorvastatin‐treated group. Atorvastatin treatment effects were consistent both in vivo and in vitro, wherein the formation of LDs and level of ROS were reduced, and the phagocytic function of microglia was restored (Figure 7r–w). Seahorse technology was used to analyze the cellular metabolic status to assess the metabolic alterations in microglia and their response to myelin phagocytosis. Atorvastatin treatment significantly reduced the OCR (Figure 7x) but significantly increased PER (Figure 7y). Furthermore, lipid metabolism in atorvastatin‐treated cells was substantially reduced, potentially owing to lipid depletion within these cells (Figure 7z). These data suggest that atorvastatin activates Pparg to improve recovery after SCI in mice.

## 3 Discussion

Tissue injury prompts the immune system to facilitate healing; optimal resolution is crucial to prevent further harm. Here, alterations in cellular communication between macrophages and microglia within the spinal cord following SCI and how these dynamics are modulated by lipid metabolism, were investigated. Following contusion, significant macrophage infiltration and enhanced interaction with microglia were observed. Furthermore, marked differences in transcriptional alterations were identified in Pparg post‐injury.

Peripherally‐derived macrophages play a pivotal role in regulating the recovery SCI by participating in debris clearance, growth factor release, and tissue remodeling.^[^ ^14^ ^]^ However, excessive inflammatory cell infiltration can have deleterious consequences. The results showed that increased macrophage infiltration in mice may contribute to slower recovery. In addition, abnormal cellular communication can disrupt native cellular functions and harm the immune microenvironment. Notably, SCI mice displayed a significant increase in intercellular communication after injury, suggesting that excessive macrophage infiltration may disrupt the original microenvironment. Transcriptome enrichment analysis revealed that the cells involved in lipid metabolism were more prevalent post‐injury. Mice with SCI accumulated more lipids following SCI.

The study identified a previously unknown microglial subtype, LBM, characterized by excessive LD accumulation and abnormal lipid metabolism. The LBM expresses more Pparg and Plin2, which may be related to their characteristics and functions.

PPARG‐deficient mice exhibited greater lipid accumulation and poorer functional recovery. This suggests that PPARG is important in lipid metabolism and functional recovery.

The heightened expression of Pparg signaling in SCI may further bolster the retention and viability of microglia. The elevated microglial counts and intracellular alterations in SCI contribute to their enduring activation.

Previous studies have indicated that elevated levels of ROS in the microglia of mice can impede functional recovery following SCI. Examining alterations in microglia metabolism that enhance ROS signaling during the SCI process could reveal potential targets for modulation, ultimately leading to improved repair mechanisms.^[^ ^15^ ^]^ Furthermore, the results showed that the aerobic and lipid metabolisms of microglia engulfing myelin were significantly induced.

The results indicate that SCI results in a large amount of macrophage infiltration, which is not conducive to recovery after injury. Lipid accumulation also affects the spinal microenvironment and is not conducive to recovery after injury. Increased Pparg expression promotes lipid metabolism and contributes to functional recovery. Atorvastatin, a commonly used drug that targets PPARG, showed beneficial effects on post‐injury recovery. It is also used on a large scale in clinics without serious side effects.

Differences remain between the simulated microenvironment of SCI in vitro and in vivo; therefore, cell metabolic changes in vivo require further study. The study did not consider complex differences in body size, physiological state, and energy expenditure between mice and humans. Therefore, more rigorous clinical trials and thorough evaluation and grouping are necessary to clarify the benefits of drugs for patients undergoing clinical use. In addition, a larger sample size is needed to describe changes in Pparg expression in patients with SCI.^[^ ^16^ ^]^

## 4 Conclusion

Overall, the study revealed changes in lipid accumulation after SCI and the positive effect of lipid metabolism interventions on functional recovery, an area that remains underexplored in CNS injuries. Future research should delve deeper into the intricate and dynamic interactions between CNS cells and their metabolism, as they may help to promote functional recovery after injury.

## 5 Experimental Section

### Patients and Clinical Data

Three patients with SCI caused by acute trauma were recruited at the First Affiliated Hospital of Nanjing Medical University between 2023 and 2024. Certified orthopedic surgeons neurologically assessed all patients. Neurological assessments, including sensory, motor, and reflex examinations below the injured segment were performed according to the American Spinal Injury Association (ASIA) Impairment Scale. The inclusion criteria were as follows: 1) trauma‐induced acute SCI between C2 and C7; 2) obvious symptoms of paraplegia or quadriplegia, assessed as ASIA grades A–B; 3) eye‐opening of 4 and verbal response of 6 according to the Glasgow Coma Scale; 4) age >18 years; 5) abnormal spinal cord signal detected using MRI; and 6) completion of effective and reliable neurological function tests. The exclusion criteria were as follows: 1) ASIA scores of C–E, 2) MRI showing no definite changes in spinal cord signaling, 3) a combination of severe craniocerebral injury and intracranial hypertension, and 4) history of tumor or autoimmune disease. Ten patients undergoing intrathecal anesthesia (with non‐neurological or neoplastic diseases) were recruited as controls. The study protocol was approved by the Ethics Committee of the First Affiliated Hospital of Nanjing Medical University (2022‐SR‐697), followed the relevant guidelines and regulations. All patients and their legal guardians provided informed consent, and third‐party assent was not allowed.

### Animals

C57BL/6J male wild‐type mice (6–8 weeks old) were purchased from GemPharmatech.

B6.129P2(Cg)‐Cx3cr1^2.1(cre/ERT2)Litt^/WganJ and B6.129‐Ppargtm2Rev/J and mice were bred in‐house; however, they were originally acquired from The Jackson Laboratory.^[^ ^17^ ^]^ C57BL/6JGpt‐Lyz2^em1Cin(CreERT2)^/Gpt, C57BL/6JGpt‐H11^em1Cin(CAG‐LSL‐tdTomato)^/Gpt, and C57BL/6JGpt‐Rosa26^em1Cin(SA‐IRES‐Loxp‐ZsGreen‐stop‐Loxp‐DTA)^/Gpt mice were acquired from The GemPharmatech Company.^[^ ^18^ ^]^ C57BL/6Smoc‐Ptprc^em1(K302E)Smoc^ was acquired from the Shanghai Model Organisms Center.

The mice were housed under a 12 h light‐dark cycle under pathogen‐free conditions. All animal procedures were approved by the Animal Committee of the First Affiliated Hospital of the Nanjing Medical University (IACUC‐2106007). Male mice were used in all the experiments.

The SCI contusion model used in the study adhered to the previously described protocols. Before the procedure, mice were anesthetized using isoflurane inhalation. After shaving the hair from the dorsal region, a midline incision was made in the skin, and the T8 laminae was excised. The spine was stabilized, and the spinal cord was subjected to contusion using a spinal impact device (68099ll device, RWD, Shenzhen, China). The impact was centered dorsoventrally at 1.0 mm, with a speed of 2 m s^−1^ and duration of 0.5 s. The control group underwent laminectomy without further SCI.

### Imaging Analysis of Humans

MRI examinations relied on a 3.0‐T superconducting MR scanner (Discovery 750, GE Healthcare, Piscataway, NJ, USA), with standard human body coil and sagittal scanning. Prior to IDEAL‐IQ, DTI was performed, the scanning parameters were: repetition time (TR)/time to echo (TE) = 4800/80 ms, b values = 800 s mm^−2^; eleven directions; thickness/spacing = 4.0/0 mm; FOV = 280 × 280 mm; TR/TE = 4800/80 ms; the number of excitations = 8; scan time = 6 min and 36 s. The DTI scan covered the C2 to T1 spinal cord and nerve roots. IDEAL‐IQ: TR = 13.2 ms, TE = 2.9 ms, flip angle = 4°, pixel size = 1.5 ×1.5 mm, slice thickness = 1.4 mm, scan time = 3 min and 21 s, and the remaining settings were the same as above. The IDEAL‐IQ scan covered the C2 to T1 spinal cord. Four group images were acquired in one scan with IDEAL‐IQ sequence: pure water, pure fat, fat fraction, and R2* relaxation rate image. After the examination was completed, the C2‐T1 spinal cord FF value were independently measured by two radiologists with at least 8 years of experience using a GE AW 4.6 post‐processing workstation. On the FF map, the sagittal midplane was selected, the region of interest (including the entire specific area of spinal cord while avoiding the vertebral body, intervertebral discs, and vertebral veins) was manually outlined to directly obtain the FF value of each SCI area, and the mean value of two personnel was used as the final value.

### Imaging Analysis of Mice

The MRI data were collected on a Bruker Pharmascan 9.4 T system (Bruker, Ettlingen, Germany). An anatomic acquisition was performed with a T2 weighted 2D RARE sequence with the following parameters: chemical fat saturation; TE, 25 ms; field of view, 26 × 26 mm; acquisition matrix, 130 × 130; number of axial slices, 20; slice thickness, 0.8 mm; RARE factor, 8; scan time, 3 min and 20 s. The acquisition was synchronized with respiration using balanced acquisitions over several respiratory periods with an effective TR of ≈2500 ms. For fat‐water separation, a 2D interleaved multiple echoes spoiled gradient echo sequence with bipolar readout gradients was used. The acquisition parameters were as follows: TR 2500 ms and number of signal averages 6. Geometric parameters were: field of view, 25× 25 mm; acquisition matrix, 180 × 180; number of slices, 20; and slice thickness, 0.6 mm; scan time, 11 min. The TR and flip angle were adjusted to minimize the T1‐related bias and work with a suitable signal‐to‐noise ratio. Phase and magnitudes images were saved systematically. The acquisition was synchronized with the respiration in animals. Prescription of localization was performed using anatomic images provided with the T2w RARE acquisition.

### Tamoxifen Treatment

Tamoxifen (1 g; HY‐13757A, MCE) was dissolved in 50 mL of corn oil (HY‐13757A, MCE) to make up a 20 mg mL^−1^ solution. Adult mice received intraperitoneal injection of 0.1 mg g^−1^ of tamoxifen solution for five consecutive days.

### Cell Depletion

Three days before operating SCI, 0.05 µg g^−1^ of diphtheria toxin was intraperitoneally injected to deplete microglia or macrophages in advance. After the injury modeling, this treatment was repeated every 3 days to sustain the depletion effect.

Mice were fed a diet containing the CSF1R inhibitor, PLX5622 (at 290 mg kg^−1^ day^−1^) for seven consecutive days to deplete microglia, starting 14 days before SCI modeling. After this period, the diet was switched to a normal diet until the end of the modeling period.

Three days before the mouse SCI modeling, an intraperitoneal injection of 250 µL of clodronate liposomes was administered to clear macrophages within the mice. Post‐SCI modeling, this treatment was repeated every 3 days to maintain the effect. The control group was simultaneously intraperitoneally injected with an equivalent volume of PBS lipomes.

### BMS Behavioral Analysis

The locomotor function of the mice was assessed using the BMS established by two trained observers in a flat, enclosed arena (diameter = 100 cm) for 4 min.^[^ ^19^ ^]^ BMS scores range from 0 to 9, with 0 indicating complete paralysis and 9 representing normal locomotion based on hind limb joint movement, weight support, plantar stepping, and coordination. The mice were evaluated for BMS scores on the first day after injury, then weekly for four weeks.

### CATWALK XT Automated Gait Analysis

The CatwalkXT automated system (Noldus) performed and analyzed motor coordination.^[^ ^20^ ^]^ In a dimly lit environment, the animals were positioned on the walkway and allowed to traverse from one end to the other. Direct contact between the paw and glass surface results in light reflection, creating illuminated footprints. A camera situated beneath the walkway captured video recordings of footprints. The images from each trial were digitized and processed using CatWalk XT 10.6 software, with a minimum threshold set at 80 (on a scale of 0–225 arbitrary units). After footprint identification and labeling, data on static and dynamic gait parameters were generated for each trial. Statistical significance was analyzed based on the mean scores from three consecutive trials (per animal/time point). Trials, where the animal stopped midway or turned around during the run, were excluded from the analysis.

### Rotarod Test

To assess motor coordination and balance in mice, rotarod (Globalebio) was used. Prior to the actual test phase, mice underwent a preparatory training regimen spanning 3 days, involving three daily practice sessions at a rotational speed of 25 revolutions per minute (rpm). During the testing phase, each mouse was individually positioned on the rotating rod within one of five designated chambers. They were allowed to remain on the rod for as long as they could maintain balance, either until they voluntarily dismounted or reached a pre‐determined maximum duration of 5 min, all at a constant speed of 25 rpm.^[^ ^21^ ^]^

### Pole‐Climbing Test

A cork ball with a diameter of 3 cm was affixed securely to the top of the pole. Mice (n = 6) were placed head‐up on the ball. Their times to turn around the ball and climb the pole were recorded. Before testing, all the mice underwent three training sessions to familiarize themselves with the entire process.^[^ ^22^ ^]^

### Perfusion and Tissue Processing

The mice were anesthetized using isoflurane (RWD) and transcardially perfused with 0.9% NaCl solution. Spinal cords were extracted, fixed in 4% paraformaldehyde (PFA) for 48 h, cryoprotected in 30% sucrose, and sectioned sagittally or coronally (10 µm) using a freezing microtome (Leica). Sections were stored at −80 °C.

### Electron Microscopy

The spinal cord was fixed with paraformaldehyde and glutaraldehyde, and the cells and liver were processed and sectioned on copper grids using a diamond knife. The grids were examined using a Hitachi 7100 electron microscope (Tokyo, Japan), and images were captured using a MegaView III digital camera (Soft Imaging System, USA). Quantification of the TEM images was performed in five randomly chosen microscopy fields.^[^ ^23^ ^]^ All experiments were repeated thrice.

### Oil Red O Staining

The sections underwent staining with Oil Red kits (Solarbio, G1261), adhering to a specific protocol. Initially, Oil Red A and Oil Red B solutions were thoroughly mixed, and the resultant mixture was subjected to filtration. Subsequently, the prepared sections were immersed in this mixture for 5 min to facilitate staining. Following staining, the sections were rinsed with double‐distilled water to remove excess stain. To further enhance the visualization, the sections were stained with hematoxylin for a brief period of 20 s, followed by another wash step. Finally, the stained sections were mounted onto glass slides and allowed to dry. For quantitative analysis, five random fields per slide were blindly photographed at a magnification of ×200 to ensure unbiased assessment.

### Immunohistochemistry and BODIPY Staining

The sections were washed thrice in PBS, followed by 1 h of blocking in PBS containing 10% donkey serum. The sections were incubated in PBS with 10% donkey serum and the following primary antibodies for 18 h at 4 °C: goat anti‐IBA1 (1:1000; Abcam, ab5076), goat anti‐PPARG (1:1000; CST, 81B8), mouse anti‐GFAP (1:1000; CST, 3670), rabbit anti‐NeuN (1:1000; CST, 24307), rabbit anti‐dMBP (1:1000; Millipore, ab5864), and rabbit anti‐PLIN2 (1:200; Proteintech, 15294‐1‐AP). After primary antibody incubation, the sections were washed thrice in PBS, and incubated in PBS with 10% donkey serum and the following secondary antibodies for 1 h at 26 °C: donkey antirabbit Alexa Fluor 555, donkey antirabbit Alexa Fluor 647, and donkey antirabbit Alexa Fluor 405 (all at 1:200 dilution; Invitrogen). Sections were washed once in PBS and incubated in PBS with BODIPY 493/503 (1:1000, Thermo Fisher Scientific) to stain LDs and Hoechst 33342 (1:2000; Thermo Fisher Scientific, 62249) for 15 min at room temperature. Four randomly selected visual fields per coverslip were photographed (×40 magnification) using a confocal scanning laser microscope (Leica) with LAX software (Leica 2023). To analyze the percentage of LD‐containing cells, the total number of Hoechst+ cells and Hoechst+ cells with BODIPY+ LDs was counted, and the percentage of BODIPY+ cells was calculated.

### ScRNA‐seq Data Processing and Identification

ScRNA‐seq was expertly executed by Singleronbio Biotechnologies (Nanjing, China).^[^ ^24^, ^25^, ^26^ ^]^ For the comprehensive analysis of the intricate single‐cell genomics data, the powerful “Seurat” software package, renowned for its ability to process and interpret high‐dimensional datasets was used. To facilitate better visualization of the single‐cell data, the Uniform Manifold Approximation and Projection technique was the dimensionality reduction method of choice. To delve deeper into the intricacies of cell‐cell communication, the “CellChat” package was used, which enabled us to quantitatively calculate and analyze the intricate interactions between individual cells.^[^ ^27^ ^]^ Additionally, locally weighted regression was used to model the expression patterns across pseudotime, considering cell fate progression, with a carefully calibrated neighbor impact factor of 0.75 to ensure accurate fitting of the data.^[^ ^28^ ^]^

### RNA‐Seq

Total RNA extraction was performed with TRIzol reagent, followed by quantifying RNA concentration using a NanoDrop 2000 spectrophotometer (Thermo Fisher Scientific). To enrich the mRNA fraction, a targeted approach was used involving magnetic beads coated with oligo(dT) probes. Subsequently, a fragmentation buffer was introduced to segment the mRNA into smaller fragments, which served as templates for the initial synthesis of cDNA strands via random hexamer priming. The second cDNA strand was synthesized by incorporating a buffer cocktail of dNTPs, RNase H, and DNA polymerase I. Subsequent purification steps, facilitated by the QiaQuick PCR purification kit, were followed by end‐repair and adding a single adenosine base (A‐tailing) to the cDNA fragments. The target fragments were then isolated through agarose gel electrophoresis and further amplified via PCR to complete the library construction process. For sequencing, the prepared libraries were subjected to HiSeq2000 (Illumina).

### DEG Analysis

PCA was performed to identify significant differences in gene expression, focusing on dimensions with *p* <0.05.^[^ ^29^ ^]^ This analysis was conducted within the R statistical environment, version 4.0.3, using the limma package. The gene expression matrix of peripheral blood monocytes from healthy individuals and patients with SCI was analyzed. Genes were screened based on a |log2FC| threshold>2 and *p* <0.05, with FDR as the correction method.^[^ ^30^ ^]^ The resulting list of up‐ and downregulated genes was visually presented through volcano plots, providing a comprehensive overview of the transcriptional alterations observed.

### Gene Function and Pathway Enrichment Analysis

To further elucidate the biological functions of the m6A‐related candidate genes, the online platform, DAVID, (https://david.ncifcrf.gov/) was used for module function and pathway enrichment analysis.^[^ ^31^ ^]^ GO analysis was conducted to annotate the genes and their products across three domains: biological process, molecular function, and cellular components. The KEGG database, which provides comprehensive information on genes, proteins, chemical compounds, and their interactions, reactions, and networks, was leveraged to annotate gene functions and metabolic pathways. In addition, all identified genes were uploaded to the STRING database to construct a protein‐protein interaction network.^[^ ^32^ ^]^

### In Vitro Phagocytosis Assay

For in vitro phagocytosis assays, cells were split into 96‐well plates at 1000 cells per well in Dulbecco's modified Eagle's medium (DMEM) + 5% fetal bovine serum (FBS), and treated with myelin and vehicle solutions or with 5% plasma and the vehicle for 24 h. Following specific treatments, 5 ng of pHRodo Red Zymosan Bioparticles (Invitrogen, P35364) in 100 µL of DMEM+5% FBS was added to each well. Phagocytosis was calculated by normalizing the red fluorescent area to the phase confluence.

### ROS Assay

To assess ROS production in primary microglia, cell homogenates were prepared and subjected to a staining procedure using CellROX Deep Red (Invitrogen) diluted in FACS buffer (1:500). This incubation was carried out at 37 °C for 30 min, followed by thorough washing with the FACS buffer. Subsequently, the intensity of CellROX Deep Red fluorescence was quantified using a flow cytometer (Beckman Coulter FC600). For a more detailed evaluation of ROS levels, cells were plated in 24‐well plates at a density of 5×10^4^ cells per well in DMEM supplemented with 5% FBS and exposed to myelin and vehicle solutions for 18 h. Subsequently, the cells were stained with CellROX Orange (Invitrogen, 1:500 dilution) in DMEM+5%FBS for 30 min at 37 °C, followed by washing with PBS. The fluorescence intensity of CellROX Orange was first visually inspected under a fluorescence microscope (Leica) and then quantitatively analyzed using flow cytometry (Beckman Coulter FC600) after detaching the cells and transferring them to FACS tubes.

### RNA Isolation and Real‐time PCR

To investigate gene expression patterns, white blood cells were isolated from patients with SCI using a Red Blood Cell Lysis Buffer (BL503A, Biosharp). Total RNA was extracted according to the manufacturer's guidelines (R6834‐02, Omega), and its quantity and quality were verified using a NanoDrop spectrophotometer (Thermo Fisher Scientific). Following RNA processing, cDNA synthesis was accomplished using the PrimeScript RT Master Mix (R222, Vazyme). Real‐time PCR was then performed on a QuantStudio 7 Flex system (Thermo Fisher Scientific) with SYBR qPCR Master Mix (Q341, Vazyme) to assess the expression levels of target genes. The ΔΔCt method was used for calculating the relative expression, normalizing to GAPDH as the housekeeping gene and using healthy controls as the reference. The specific primers used for this analysis were: GAPDH forward primer, AGGTCGGTGTGAACGGATTTG; GAPDH reverse primer, TGTAGACCATGTAGTTGAGGTCA; PPARG forward primer, TCGCTGATGCACTGCCTATG; and PPARG reverse primer, GAGAGGTCCACAGAGCTGATT. ABCA1 forward primer, GCTTGTTGGCCTCAGTTAAGG; and ABCA1 reverse primer, GTAGCTCAGGCGTACAGAGAT. ABCG1 forward primer, CTTTCCTACTCTGTACCCGAGG; and ABCG1 reverse primer, CGGGGCATTCCATTGATAAGG.

### Western Blotting

To prepare spinal cord tissue (50 mg) or cells for protein analysis, they were mechanically homogenized in 1 mL of RIPA buffer fortified with 1% protease inhibitor complex (Sigma–Aldrich, P2714). The homogenates were maintained on ice for 30 min to ensure complete cell lysis. Subsequently, the protein extracts underwent separation via 10% sodium dodecyl sulfate‐polyacrylamide gel electrophoresis (SDS‐PAGE) and then subjected to immunoblotting. During SDS‐PAGE, an equal quantity of protein (50 µg) was loaded into each well and transferred onto PVDF membranes (Sigma‐Aldrich, IPVH00010). Before antibody probing, the membranes were blocked with a solution of Tris‐buffered saline (TBST, Sigma‐Aldrich, T6664) containing 0.05% Tween 20 (Solarbio, T8220) and 5% nonfat milk for 2 h. Following three washes with TBST (5 min each), the membranes were incubated with primary antibodies against PPARG (CST, 81B8) and GAPDH (CST, 2118), diluted between 1:200 and 1:1000, to detect the target proteins. After another series of three washes, the membranes were exposed to secondary antibodies conjugated with horseradish peroxidase, diluted 1:7500, for 2 h at 26 °C. After thorough washing with TBST, the membranes were illuminated using an ECL chemiluminescent kit (ECL‐plus, Thermo Fisher Scientific, 34095), enabling the capture of band images by a camera and their subsequent storage on a computer. The ImageJ densitometry software (v1.41) facilitated the transfer of these images into analyzable data, with at least three images used for each data point.

### Flow Cytometry Analysis

Cells were pretreated with myelin or other treatment for 12–24 h for the assay. The cells were then trypsinized and resuspended in 1 mL chilled PBS (Hyclone, SH30256.01). The cell suspensions were held on ice and analyzed using flow cytometry (Beckman Coulter, FC600). Data processing was performed using FlowJo software (v10, Tree Star).

### Seahorse Mitostress Assays

Microglia cells were transferred into individual wells of a Seahorse XF Islet Capture Plate, containing 80 µL of Seahorse XF DMEM media enriched with 17.5 mm glucose, 0.5 mm pyruvate, and 2 mm glutamine. Upon transfer, the cells were washed twice with the media and incubated for 1 h at 37 °C in a non‐CO_2_ incubator before the Mitostress Assay. This assay was specifically designed to measure four cycles of basal respiration, five cycles after oligomycin injection (Seahorse, 1.5 µm), five cycles after FCCP injection (Seahorse, 2 µm final concentration), and five cycles after Rotenone+AntimycinA injection (Seahorse, 0.5 µm). In addition, the rate of glycolysis was evaluated at specified final concentrations of 0.5 µm rotenone/antimycin A 25 mm 2‐DG.

During the fatty acid metabolism test, the culture medium in the Seahorse assay plates was replaced with substrate‐limited medium for fatty acid oxidation and incubated for 30 min. Etomoxir (4 µm final, Agilent) was administered to half of the wells in each group and allowed to incubate for 15 min. Subsequently, palmitate‐BSA or BSA control was added, and analysis was initiated using the XF assay. Mitochondrial function was evaluated as described in Figure 3a at the following final concentrations: 1.5 µm oligomycin, 2 mm FCCP, and 0.5 µm rotenone/antimycin A.^[^ ^33^ ^]^

Upon completion, the cell count was measured using a Cytation system (BioTek). Three wells (n = 3) were used in each experiment. The OCR and ECAR values were precisely calculated using Wave Desktop Software (version 2.6.3). Basal respiration, ATP production, respiratory capacity, and respiratory reserves were determined according to the manufacturer's protocol.

### Molecular Docking

The amino acid sequence of PPARG (P37231) was retrieved from UniProt, and the Swiss Model was used to identify an appropriate structural template, construct a homology model, and assess its quality. Using PyMOL, a molecular docking model was devised between GPS and the 3D structure of the PPARG homology model. The refined protein structure was leveraged to pinpoint crucial amino acids within the predicted binding site. Following energy minimization of the ligand, interactive docking of GPS conformers into the selected active site was conducted using PyMOL. Binding energies were assigned to the docked compounds, contingent upon their specific binding modes at the site. A protein‐ligand interaction fingerprint was implemented to classify the types of interactions occurring.

### SPR Analysis

SPR experiments were conducted on a Biacore 8 K instrument (GE Healthcare). Purified PPARG protein (200 mg mL^−1^, pH 4.5) was immobilized on a Series S Sensor Chip (GE Healthcare) adhering to standard amination protocols, with PBS containing 5% DMSO serving as the running buffer. GPS solutions were prepared via serial dilutions of the stock in the running buffer, and seven concentrations were simultaneously injected at 65 mL min^−1^ for 60 s during the association phase at 25 °C. Blank sensorgrams were subtracted to yield final graphs. Data acquisition and analysis were facilitated by the Biacore 8 K Manager software, aiming to fit an appropriate binding model and determine the equilibrium dissociation constant (Kd).

### MST

Additionally, Kd measurements were performed using a Monolith NT.115 instrument (NanoTemper Technologies). PAQR3 was fluorescently labeled as per the manufacturer's instructions. GPS was diluted to specified concentrations (5–1 mmol L^−1^) and incubated with 0.85 mg mL^−1^ of purified, labeled PAQR3 protein in running buffer (50 mmol L^−1^ Tris‐HCl, 100 mmol L^−1^ NaCl, pH 7.5) for 15 min. The samples were loaded into NanoTemper glass capillaries, and MST was executed with 80% LED power and 80% MST. Duplicate measurements were used to calculate Kd values employing the mass action equation and NanoTemper software.^[^ ^34^ ^]^

### CCK8 Assay

The cells were plated in a 96‐well plate (3 × 10^3^ cells well^−1^) in 100 µL DMEM containing 10% FBS and 1% antibiotics. On the indicated day, 10 µL of CCK‐8 reagent (C0037; Beyotime, China) was added and incubated for 2 h. Absorbance (450 nm) was measured using a spectrophotometer.

### Lipid‐Targeted Metabolomics Analysis

Lipids from the spinal cord were extracted using methyl tert‐butyl ether/methanol (5:1 ratio) and spiked with the internal standards. The samples were centrifuged, and the supernatants were dried using a vacuum concentrator. The resulting pellet was resuspended in dichloromethane/methanol/H_2_O (60:30:4.5, v/v). The mixed solution was centrifuged, and the supernatant was used for liquid chromatography‐tandem mass spectrometry analysis (Biotree Biotech. Co.).^[^ ^35^ ^]^

### CUT&Tag Analysis

The Hyperactive Universal CUT&Tag Assay Kit (Vazyme, TD904) and TruePrep Index Kit V2 (Vazyme, TD202) were used. Cells were gathered and rinsed with 500 µL of wash buffer, subsequently bound to ConA beads for 10 min at 26 °C. Overnight incubation with the primary antibody (anti‐PPARG, IgG used as control) was conducted at 4 °C. Subsequently, secondary antibodies were introduced, and the cells were incubated for 60 min at 26 °C. Following three washes with DIG wash buffer, the cells were incubated with 0.04 µm pA/G–Tnp for 60 min at 26 °C. Similarly, after three washes with DIG 300 buffer, the cells were resuspended in fragmentation buffer and incubated at 37 °C for 1 h. Post‐fragmentation, proteinase K, LB buffer, and DNA extraction beads were added to the mixture. Following a 10‐min incubation at 55 °C, the beads were rinsed with Buffer WA and Buffer WB. DNA elution was performed at 26 °C for 5 min using 22 µL of H_2_O. The TruePrep Index Kit V2 for Illumina (Vazyme, TD202) was used to construct the CUT&Tag libraries according to the manufacturer's guidelines.^[^ ^36^ ^]^

### Statistical Analysis

All results were presented as mean ± SEM and statistical significance was set at *p <*0.05. Statistical analyses were performed using GraphPad Prism, v9.0 (GraphPad, San Diego, CA, USA). For comparisons between groups, an unpaired Student's *t*‐test was used for normally distributed parameters, whereas the Mann–Whitney U test was used for non‐normally distributed parameters. For experiments involving multiple comparisons, a one‐way analysis of variance (ANOVA) was performed, followed by either Tukey's post‐hoc test or Brown–Forsythe and Welch ANOVA tests, accompanied by Dunnett's T3 multiple comparison test for normally distributed parameters. In the case of non‐normally distributed parameters with multiple datasets, the Kruskal–Wallis test was applied. Correlations were assessed using Spearman's correlation analysis. All the results are presented as mean ± standard deviation. Data were analyzed using Student's *t*‐test or one‐way analysis of variance with GraphPad Prism 9. Differences between groups were considered statistically significant when the two‐sided p‐values were <0.05.

### Ethics Approval Statement

The study protocol was approved by the Ethics Committee of the First Affiliated Hospital of Nanjing Medical University (2022‐SR‐697). All animal procedures were approved by the Animal Committee of the First Affiliated Hospital of Nanjing Medical University (IACUC‐2106007).

### Patient Consent Statement

All patients and their legal guardians provided informed consent.

## Conflict of Interest

The authors declare no conflict of interest.

## Data Availability

The data that support the findings of this study are available from the corresponding author upon reasonable request.
